# Alterations in short-term blood pressure variability related to disease severity and autonomic symptoms in myasthenia gravis patients

**DOI:** 10.1007/s10072-023-06927-1

**Published:** 2023-06-28

**Authors:** Monika Zawadka-Kunikowska, Łukasz Rzepiński, Mirosława Cieślicka, Jacek J. Klawe, Małgorzata Tafil-Klawe

**Affiliations:** 1 Department of Human Physiology, Nicolaus Copernicus University, Ludwik Rydygier Collegium Medicum in Bydgoszcz, Karłowicza 24, 85-092 Bydgoszcz, Poland; 2 Sanitas—Neurology Outpatient Clinic, Dworcowa 110, 85-010 Bydgoszcz, Poland; 3 Department of Neurology, 10th Military Research Hospital and Polyclinic, 85-681 Bydgoszcz, Poland; 4 Department of Hygiene, Epidemiology, Ergonomy and Postgraduate Education, Nicolaus Copernicus University in Torun, Ludwik Rydygier Collegium Medicum in Bydgoszcz, M. Sklodowskiej-Curie 9, 85-094 Bydgoszcz, Poland

**Keywords:** Myasthenia gravis, Blood pressure variability, Autonomic dysfunction, Postural tachycardia syndrome, Orthostatic intolerance, COMPASS-31 scale

## Abstract

**Objectives:**

We aimed to evaluate beat-to-beat blood pressure variability (BPV) during head-up tilt test (HUTT) in patients with mild and moderate myasthenia gravis (MG) compared to healthy controls (HCs), and its association with the severity of autonomic symptoms.

**Methods:**

A total of 50 MG patients and 30 HCs were evaluated. Patients were stratified into 2 groups regarding Myasthenia Gravis Foundation of America (MGFA) classification: mild (I,II MGFA), moderate form (III MGFA). Autonomic symptoms were assessed by COMPASS-31 questionnaire. Cardiovascular parameters, indices of very short-term systolic (SBPV), and diastolic blood pressure (BP) variability (DBPV) were assessed at rest, and during HUTT.

**Results:**

Moderate MG patients were characterized by an overall shift of sympathovagal balance toward sympathetic predominance, either at rest and during HUTT, as well as lower values of high frequency (HFnu) of DBPV during HUTT, compared to HCs and mild MG. Similarly, moderate MG showed higher resting low frequency (LFnu) of DBPV (*p*=0.035), higher COMPASS-31 score (*p*=0.031), and orthostatic intolerance sub-score (*p*=0.019) than mild MG patients. Compared to HCs, mild MG patients showed lower Δmean BP (*p*=0.029), Δdiastolic BP (*p*=0.016). Autonomic symptoms were associated with lower BP values, at rest and during HUTT, and lower LF BPV parameters during HUTT.

**Conclusion:**

MG patients present significant alterations in BPV, both at rest and in response to orthostatic stress, which are related to autonomic symptoms and disease severity. This study confirms the importance of monitoring BPV when evaluating cardiovascular autonomic function and its evolution over the course of MG disease.

## Introduction

Myasthenia gravis (MG) is a rare, autoantibody-mediated neuroinflammatory disease caused by the production of pathogenic immunoglobulin G autoantibodies targeting neuromuscular junction proteins. MG is a heterogeneous disease with significantly disabling clinical consequences including fluctuating muscular fatigability, localized or general muscle weakness, dyspnea, and impaired functional mobility [1, 2]. Acetylcholine receptor (AChR) autoantibodies and muscle-specific kinase (MuSK) antibodies are still considered the most specific pathogenic factors leading to the onset of MG [3].

In the past few decades, there has been a growing debate on overlooked non-motor symptoms such as cognitive, sleep, autonomic, and sensory disturbances of the clinical presentation of MG [4, 5]. Recently, studies have demonstrated that cardiac autonomic dysfunction (CAD) in MG is much more frequent than previously thought. Furthermore, studies using cardiovascular challenges suggest that autonomic dysregulation contributes to the higher blood pressure (BP) and heart rate (HR) fluctuations and involve abnormal orthostatic responses [6]. CAD is often characterized by reduced baroreflex sensitivity, decreased parasympathetic activity, and relative sympathetic hyperactivity in different MG patients subgroups [7-13]

While Ewing’s battery and Composite Autonomic Severity Score (CASS) have been widely used as standard tests for screening autonomic dysfunction [14, 15], less is known regarding the assessment of very short-term beat-to-beat blood pressure variability (BPV) in patients with MG, indicating possible changes in these parameters [15]. Evaluation of BPV is increasingly recognized as sensitive assessment of cardiovascular regulation that provides useful information about alterations in autonomic activity such as abnormal sympathetic modulations and baroreflex function [16]. Conventional methods to assess BPV include measurement of different time periods: very short-term (beat-to-beat), short-term (within 24 h, minute-to-minute, hour-to-hour, and day-to-night), mid-term (day-to-day), and long-term (visit-to-visit over weeks, months/years) [16]. The use of self-reported questionnaires, in particular Composite autonomic symptom score 31 (COMPASS-31), has been widely used to recognize global ANS symptoms in patients with neurological disorders [17, 18].

Previous studies did not take into account beat-to-beat BPV and its implication in the context of severity of MG. Thus, considering bidirectional interactions between the immune and autonomic systems we hypothesized that individuals with greater disease severity would be more likely to have orthostatic intolerance symptoms and greater alterations in BPV in response to orthostatic challenge. We aimed to evaluate beat-to-beat blood pressure variability (BPV) during head-up tilt test (HUTT) in patients with mild and moderate MG as compared to healthy controls (HCs), and its association with the severity of autonomic symptoms.

## Materials and methods

### Participants

In this cross-sectional study, clinically stable patients with MG were enrolled from an outpatient clinic (Sanitas, Bydgoszcz, Poland) between December 2018 and December 2022. The Bioethical Committee of Collegium Medicum in Bydgoszcz, Nicolaus Copernicus University in Torun (No. 747/2017) approved the study protocol, and written informed consent for study participation was obtained from all participants. All patients underwent a detailed clinical and neurological examination. Diagnosis of MG was confirmed by neurologists with expertise in neuromuscular disorders on the basis of fatigable limb, bulbar, respiratory, or ocular weakness, and either abnormal electrophysiology (repetitive nerve stimulation or single fiber electromyography) or serology (positive AChR or MuSK antibodies).

Inclusion criteria comprised a previous diagnosis of MG; no exacerbation of symptoms at the time of assessment; age at least 18 years; no medical history of other disabling pathologies; no neurological diseases apart from MG; absence of previous psychiatric disorders; ability to estimate self-reported scores independently; and absence of mechanical ventilation (MGFA clinical classification = 5). Exclusion criteria for MG patients and HCs comprised age younger than 18 years; the presence of major concurrent illness (respiratory involvement or in state of MG crisis); diabetes, hypertension, or any other disease that might affect the ANS; treatment with beta-blockers, antihypertensive drugs. HCs were identified from the local community of Bydgoszcz, Poland. HCs were excluded if they had experienced central/peripheral nervous system lesions and any other disease known to affect the ANS.

Patients with ocular as well as generalized myasthenia gravis were included in the study. Clinical severity was determined using Myasthenia Gravis Foundation of America (MGFA) classification into I–V [19, 20]. Class I indicates pure ocular weakness, class II mild-generalized weakness, class III moderate generalized weakness, class IV severe generalized weakness, and class V intubation/myasthenic crisis. Within generalized MG (II–IV), patients are classified into subgroups according to predominance of muscle weakness: (a) predominant limb/axial muscles involvement*;* (b) predominant bulbar-oropharyngeal/respiratory muscles involvement [19, 20]. Baseline demographic and clinical characteristics were obtained from medical records. In the MG group, disease duration, MG clinical severity, clinical parameters (presence of thymectomy, AChR antibodies, anti-MUSK antibodies, disease duration, age at onset and EMG results (repetitive and single fiber)) were also collected [1]. The patients were stratified into two groups regarding MGFA classification: mild form (MGFA I, II) and moderate form with MGFA III of MG. Serum levels of AChR antibodies were detected with enzyme-linked immunosorbent assay (ELISA). IgG4 antibodies against MuSK were measured by ELISA in subjects lacking anti-AChR antibodies. Myasthenic exacerbation was defined as the clinical deterioration of previously reported muscle weakness lasting more than 24 h unrelated to fever and/or infection, resulting in an increase in the MGFA classification by at least one class. The worsening of symptoms within the last 30 days was considered a single exacerbation [21]. Thymic pathology was assessed in accordance with the CT imaging findings and available histology findings.

### Autonomic testing

Continuous monitoring of heart rate (HR) and beat-to-beat blood pressure (BP) was recorded noninvasively with a Task Force Monitor System (TFM, CNSystems, Medizintechnik, Graz, Austria). All investigations were performed in our laboratory under standardized conditions meeting criteria for functional testing of the ANS, in a quiet, darkened, and temperature-controlled room (22 ± 1 °C). Data were collected between 8 and 12 AM. TFM is a reliable and reproducible method of non-invasive measurement of autonomic function. Subjects were asked to eat a light breakfast, refrain from drinking coffee, smoking, alcohol, and exercise on the day of testing [22].

All subjects underwent continuous HR, BPV monitoring during at least 10 min supine rest and during the head-up tilt test (HUTT), using a 70° angle of tilt for 7 min. The HR was measured with an electrocardiogram (ECG), while BP was measured through cuffs on the index and middle fingers of right hand that was compared automatically to the oscillometric blood pressure measured on the contralateral arm [23]. Respiration rate was derived from the thoracic impedance [24].

The TFM software evaluated power spectral analysis for blood pressure variability (BPV) via the adaptive autoregressive modeling (AAR) proposed by Bianchi et al. using a recursive least-squares algorithm [24].

The frequency components of BPV included oscillations at very low frequency (VLF; <0.04 Hz), low frequency (LF;0.04–0.15 Hz), high frequency (HF; 0.15–0.40 Hz), total power spectral density (PSD; <0.40 Hz), and LF/HF ratio ) from beat-to-beat BP and heart rate monitoring. [22, 24].

Postural orthostatic tachycardia syndrome (POTS) was defined as a persistent increase in heart rate of at least 30 beats per minute within 10 min of upright tilt, in the absence of either classical or delayed orthostatic hypotension. The diagnosis of orthostatic hypotension (OH) was made if there was as a drop in blood pressure (BP) of at least 20 mm Hg for systolic blood pressure (sBP) or 10 mm Hg for diastolic blood pressure (dBP) within 3 min during a head-up tilt test [16].

### COMPASS 31 questionnaire

Composite Autonomic Symptom Scale 31 (COMPASS 31) questionnaire was used to measure the severity of symptoms of AD. The COMPASS 31 consists of 31 items in 6 domains orthostatic intolerance (OI), vasomotor, secretomotor, gastrointestinal, bladder, and pupillomotor. The total weighted scores of COMPASS 31 range from 0 to 100, and the orthostatic intolerance weighted sub-scores range from 0 to 5, with a higher score indicating greater autonomic dysfunction [17, 18].

### Statistical analysis

In our study, BPV data were exported from the TFM program into Microsoft Excel for further analysis. All data were then imported into Statistica 13. The AAR model may produce outliers; thus, all BPV beat-to-beat data were filtered using Grubbs’s test for outliers’ elimination. This method of filtering is well-documented and has a strong mathematical background) [25]. The data were analyzed using the StatSoft, version 13.3. All data were presented as mean ±SD or median and (25^th^–75^th^ percentile) based on variable distribution. Categorical variables are presented as absolute (*n*) and relative frequency (%). The normal distribution of the study variables was verified with Shapiro-Wilk test. Differences in the distribution of categorical variables were determined with Chi-square test or Fisher’s exact test, while the differences in continuous variables were determined with the use of Student *t* or nonparametric Mann-Whitney test. Multiple comparisons were performed by parametric analysis of variance (ANOVA), followed by the Bonferroni post hoc test or by non-parametric Kruskal-Wallis rank-sum test. A *p* value of less than 0.05 was considered statistically significant.

## Results

### Demographic and clinical data

Baseline demographic data and subjects characteristics are shown in Table 1. A total of 50 (42 female; age range; 19–69 years) MG patients and 30 HCs met all inclusion criteria and none of the exclusion criteria. All patients were clinically stable and received pyridostigmine (mean dose 240 mg/day). A total of 58% of patients received corticosteroids (prednisone 30 mg/day), and 28% of patients required immunosuppressive agents (9 azathioprine, mean dose 150 mg/day and 5 mycophenolate mofetil, mean dose 1000 mg/day). MGFA class at the moment of testing was available for all patients. Out of 50 MG patients, moderate and mild form of the MG disease were observed in 36% and 64% (18% and 46%) patients, respectively. There were no significant differences between mild (mean age 40.1±11.3; 25 female, 7 male), moderate (43.2±8.8; 17 female, 1 male) MG groups, and the HCs (mean age 39.4±9.0; 21 female, 9 male) with respect to age (*p*=0.268) and sex (*p*=0.133) distribution. Thymic pathology was detected in 21 (59.5%) of mild and 8 (44.4%) of moderate MG patients, and fifteen of them underwent thymectomy. Histopathological evaluation revealed thymoma in one case and thymic hyperplasia in the others. There were significant differences between the mild and moderate MG group with respect to percentage on administration of corticosteroids (46.9% vs 69.8%, *p*=0.033) and immunosuppressive agents (12.5% vs 55.6%, *p*=0.001), respectively (Table 1).

**Table 1:** Demographic and clinical data of the study participants

	Total	Mild MG	Moderate MG	*p* value
Number of subjects	50	32	18	
Sex, female *n* (%)	42 (84)	25 (78.1)	17 (94.4)	0.130
Age, mean (years)	41.2±10.5	40.1±11.3	43.2±8.8	0.209
Age at first manifestation, mean, (years)	34.09±11.4	33.5 (12.4)	35.1±9.6	0.524
Early-onset MG (< 50 years)	46 (92.0)	29 (90.6)	17 (94.4)	0.632
Disease duration (years), mean (range)	7.1±7.2	6.5±7.6	8.1 (6.4)	0.113
Seropositivity to AChR antibodies, *n* (%)	28 (56.0)	20 (62.5)	8 (44.4)	0.298
Seropositivity to MuSK antibodies, *n* (%)	3 (6.0)	1 (3.1)	2 (11.1)	0.253
Type of MG, *n* (%)				0.032
Ocular	7 (14.0)	7 (21.9)	0 (100)	
Generalized	43 (86.0)	25 (78.1)	18 (100)	
Thymectomy, *n* (%)	15 (30.0)	12 (37.5)	3 (16.7)	0.151
Severity of disease at the moment of testing (MGFA, %)				
Class 0	0 (0)	0 (0)	0 (0)	
Class I (ocular)	8 (16.0)	8 (25.0)	0 (0)	
Class IIa	24 (48.0)	24 (75.0)	0 (0)	
Class IIIa	18 (36.0)	0 (0)	18 (100)	
Histology changes, *n* (%)				
Thymic pathology	29 (58.0)	21 (65.6)	8 (44.4)	0.079
Thymoma	1 (2.0)	1 (3.1)	0 (100)	0.433
Unknown	2 (4.0)	2 (6.3)	0 (100)	
Type of treatment, *n* (%)				
Pyridostygmine	50 (100)	32 (100)	18 (100)	1.00
Pyridostygmine alone	21 (42)	17 (53.3)	4 (22.2)	0.032
Pyridostygmine + cortisteroids	29 (58.0)	15 (46.9)	14 (77.8)	0.033
Pyridostygmine+Corticosteroids+Azathioprine/Mycophenolate	14 (28.0)	4 (12.5)	10 (55.6)	0.001
COMPASS 31 Total weighted score, median [range]	31.8 [18.8–49.4]	28.9 [11.9–40.4]	38.6 [27.9–55.1]	0.02
COMPASS 31 Orthostatic weighted sub-score, median [range]	20.0 [0–24]	14.0 [0–24]	20.0 [20.0–28.0]	0.016

*MG* myasthenia gravis, *HCs* healthy controls, *AChR* acetylcholine receptor, *MGFA* Myasthenia Gravis Foundation of America, *MuSK* muscle-specific kinase receptor COMPASS 31 Composite Autonomic Symptom Scale 31

### Cardiovascular and autonomic data assessment: mild MG, moderate MG, and the control group comparisons

**Fig. 1:**
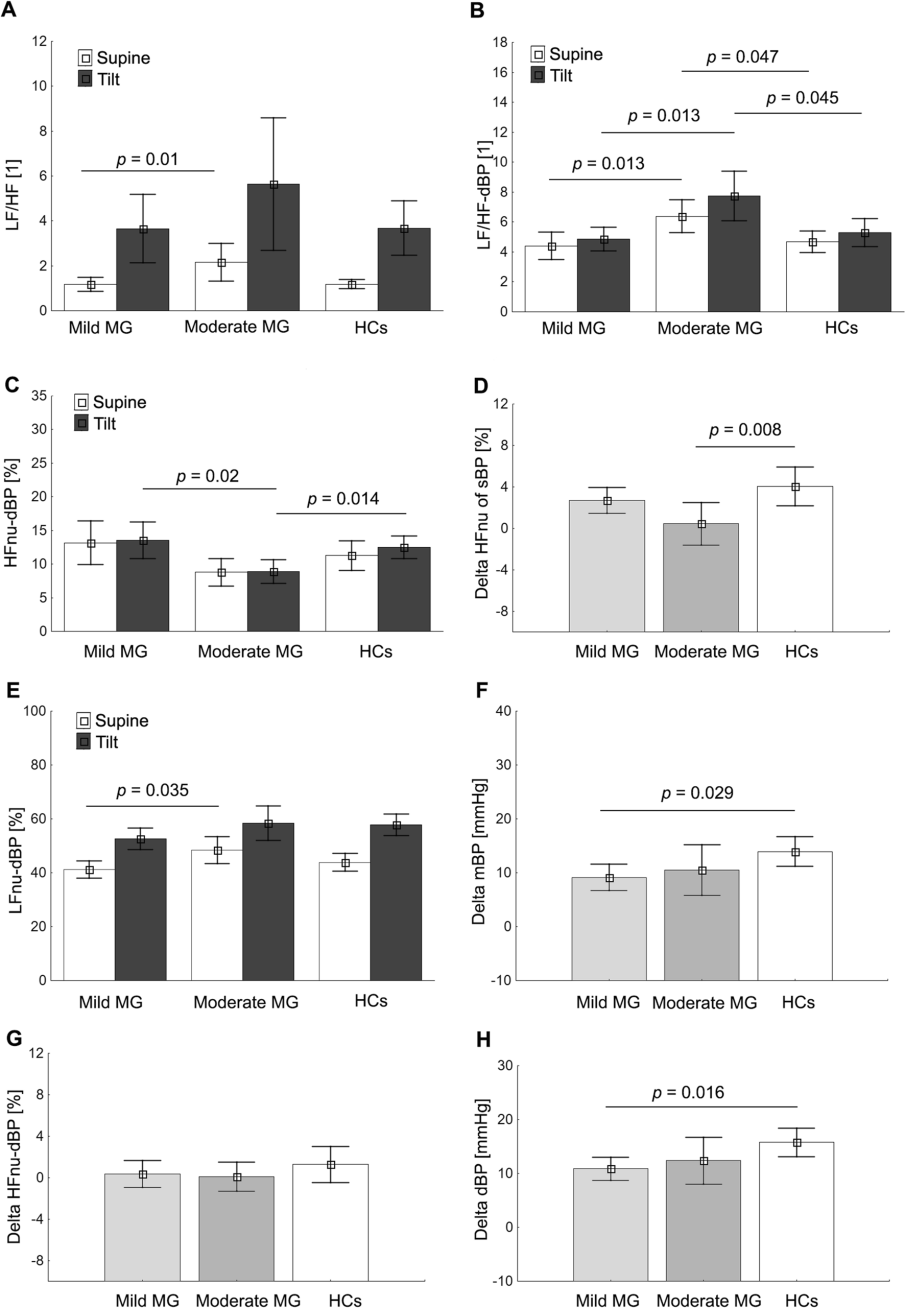
Mean±SD values of LF/HF, ratio between low and high band for heart rate and blood pressure variability (**A**); LF/HF-dBP, ratio between low and high band for diastolic blood pressure variability (**B**); HFnu-dBP, high frequency of diastolic blood pressure variability in normalized units (**C**); Delta HFnu-sBP, Delta high frequency of systolic blood pressure variability in normalized units (**D**); LFnu-dBP, low frequency of diastolic blood pressure variability in normalized units (**E**); Delta mBP, Delta mean blood pressure (**F**); Delta HFnu-dBP, Delta High frequency of diastolic blood pressure variability in normalized units (**G**); Delta dBP, delta diastolic blood pressure in MG subgroups; respectively compared to HCs

The majority (94%) of MG subjects had normal heart and blood pressure responses to standing. Only two MG patients (MGFA IIa) had POTS and one (MGFA IIIa) OH. At rest, moderate MG patients showed significantly higher LFnu-dBP, sympathovagal balance ratio in the form of LF/HF and LF/HF-dBP when compared to mild MG as well as higher values of LF/HF-dBP compared to HCs (*p*=0.047) (Fig. 1, Table 2). In contrast, no significant differences were observed between the MG and control groups in HR and BP parameters (*p>*0.05). When comparing orthostatic response, moderate MG group showed significantly lower values of HFnu-dBP and LF/HF-dBP during HUTT when compared to mild MG (*p*=0.02, *p*=0.013) and HCs (*p*=0.014, *p*=0.045) respectively, while patients with mild MG had no significant differences compared to HCs (*p*>0.05). Furthermore, compared to HCs, mild MG patients showed significantly lower post-tilt changes in ΔmBP and ΔdBP (Fig. 1). Similarly, moderate MG group showed lower post-tilt changes in ΔHFnu-sBP than the HCs group, although mean BP profiles did not differ significantly. Moreover, at rest and during tilt, HCs had significantly lower respiratory rate (15.6±2.4 vs 14.7±2.9 bpm, when compared to mild MG (17.8±1.8 vs 18.3±2.1 bpm, *p*=0.002) as well as those with moderate MG (18.3±2.3 19.1±1.9 bpm, *p*<0.001) patients, respectively.

**Table 2:** Mean ± standard deviation of resting and during tilt test hemodynamic parameters for mild MG, moderate MG patients, and HCs

Group	Mild MG	Moderate MG	HCs	Mild MG	Moderate MG	HCs	Mild MG	Moderate MG	HCs
	Rest			70° tilt			Δ (Delta tilt-supine)		
Cardiovascular measures									
HR [1/min]	62.8±7.7	67.2±6.9	65.7±9.9	77.2±10.6	77.0±7.9	79.5±10.1	14.4±8.2	9.8±6.2	13.8±6.3
sBP [mmHg]	114.4±11.1	111.2±12.3	110.2±11.8	122.2±11.0	120.8±9.5	122.7±11.2	7.6 ±9.7	9.6±12.5	12.5±9.4
dBP [mmHg]	72.2±8.5	73.3±8.8	69.5±9.6	83.1±9.3	85.7±9.3	85.3±1.6	**10.9±6.1**	12.4±9.3	15.8±7.2*
mBP [mmHg]	89.9±8.8	89.9±9.7	86.8±10.4	99.0±9.5	100.4±8.5	100.7±1.6	**9.1±6.9**	10.5±10.0	13.9±7.6*
Systolic blood pressure variability (SBPV)									
LFnu-sBP [%]	38.0±10.7	43.9±9.6	40.0±8.1	49.7±12.2	54.0±14.1	54.0±10.8	11.7±10.2	10.1±11.1	14.0±11.8
HFnu-sBP [%]	17.6±13.7	13.0±5.6	12.9±6.0	20.3±13.2	13.5±5.2	16.9±6.1	2.7±3.5	**0.4±4.3**	4.0±5.1**
LF-sBP [mmHg^2^]	6.2±5.7	5.8±2.7	5.2±2.9	5.6±5.0	5.2±2.3	5.2±3.6	−0.6±2.6	−0.6±2.4	−0.0±2.3
HF-sBP [mmHg^2^]	2.8±3.5	1.7±0.9	1.7±1.5	2.2±2.1	1.4±0.9	1.5±1.0	−0.6±2.0	−0.3±0.8	−0.2±0.8
PSD-sBP [mmHg^2^]	17.1±19.5	13.8±7.5	13.4±7.1	11.1±9.3	10.0±4.1	9.3±4.8	−6.0±2.0	−3.8±6.3	−4.1±4.5
LF/HF-sBP [1]	3.2±2.1	4.1±2.3	3.7±1.6	3.4±2.0	4.6±2.1	3.8±2.0	0.2±0.9	0.5±1.5	0.1±2.1
LF/HF [1]	**1.2±0.9**	2.2±1.7*	1.2±0.6	3.7±4.2	5.6±5.9	3.7±3.2	2.5±3.9	3.4±5.5	2.5±2.9
Diastolic blood pressure variability (DBPV)									
LFnu-dBP [%]	**41.2±9.2**	48.4±10.7*	43.9±8.9	52.7±11.2	58.4±13.6	57.8±10.9	11.5±12.5	10.0±11.9	13.9±11.1
HFnu-dBP [%]	13.1±9.1	8.8±4.3	11.3±6.1	**13.5±7.7**	8.9±3.7*	12.5±4.6*	0.4±3.7	0.1±2.9	1.2±4.8
LF-dBP [mmHg^2^]	3.8±2.7	4.4±2.9	3.7±2.2	3.6±2.2	4.0±2.0	3.8±3.0	−0.2±1,5	−0.4±2.6	0.1±1.6
HF-dBP [mmHg^2^]	1.3±1.8	0.9±0.9	0.9±0.6	1.0±1.0	0.6±0.5	0.8±0.3	−0.3±0.9	−0.3±0.8	−0.1±0.5
PSD-dBP [ms^2^]	9.3±7.0	9.3±5.5	8.7±4.6	6.9±4.3	6.9±3.3	6.5±4.1	−2.4±4.2	−2.4±4.7	−2.2±2.4
LF/HF-dBP [1]	4.4±2.6*	**6.4±2.3**	3.7±2.0*	4.9±2.3*	**7.7±3.5**	5.3±2.6*	0.4±1.9	1.3±2.1	0.6±2.4

Statistically significant differences (*p <0.05, **p<0.01) are indicated in bold text

*MG* myasthenia gravis, *HCs* healthy controls, *HR* heart rate, *sBP* systolic blood pressure, *dBP* diastolic blood pressure, *mBP* mean blood pressure, *LFnu-dBP* low frequency of diastolic blood pressure variability in normalized units, *HFnu-dBP* high frequency of diastolic blood pressure variability in normalized units, *PSD-dBP* power spectral density of diastolic blood pressure variability, *LF/HF-dBP* ratio between low and high band for diastolic blood pressure variability, *LFnu-sBP* low frequency of systolic blood pressure variability in normalized units, *HFnu-sBP* high frequency of systolic blood pressure variability in normalized units, *PSD-sBP* power spectral density of systolic blood pressure variability, *LF/HF-sBP* ratio between low and high band for systolic blood pressure variability, *nu* normalized values

### COMPASS-31 scale and relationship between autonomic symptoms and cardiovascular, BPV parameters, and clinical outcomes

**Fig. 2:**
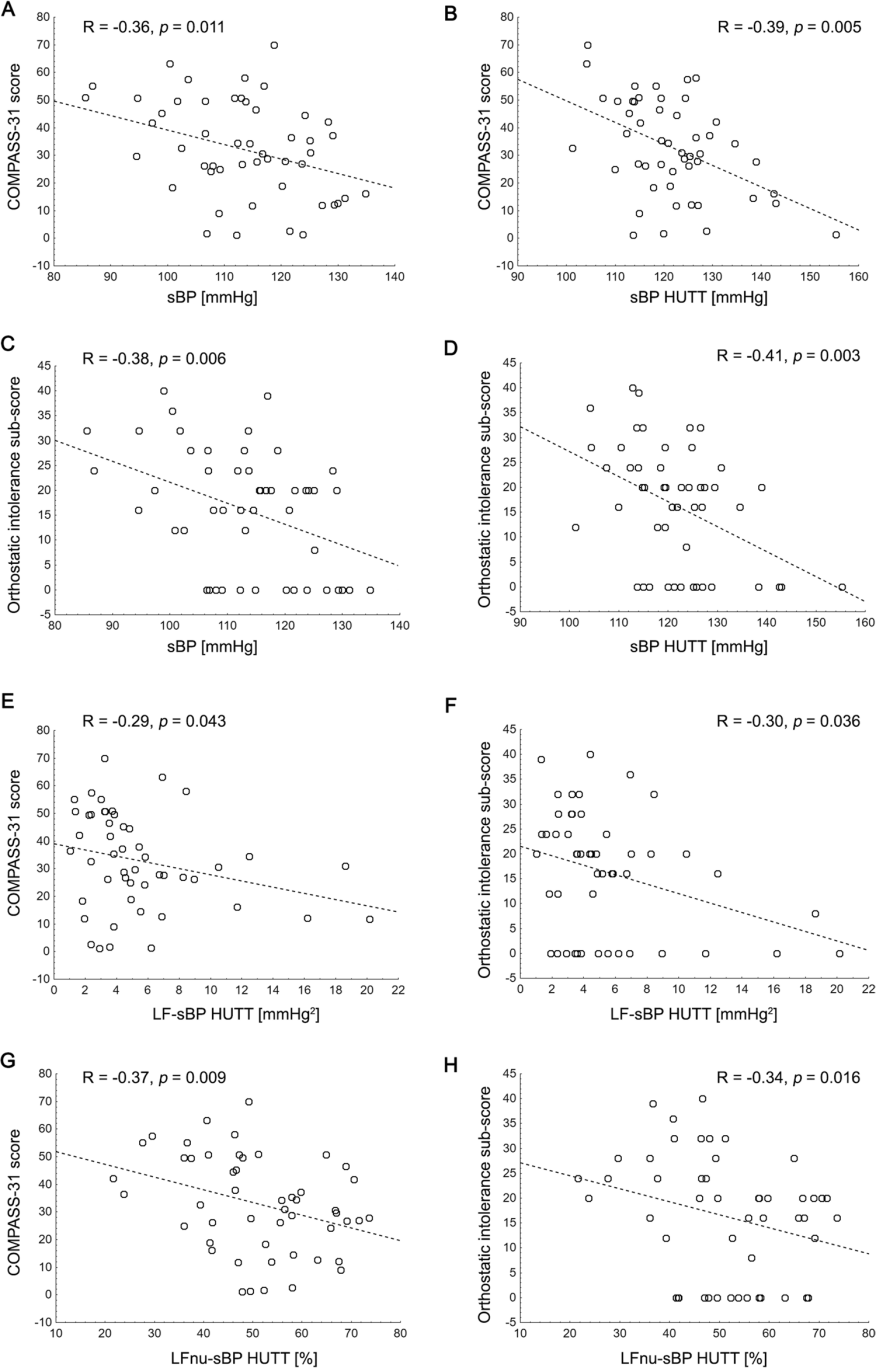
Correlation between the total scores assessed with COMPASS 31 questionnaire with sBP, systolic blood pressure (**A**–**B**); orthostatic intolerance and sBP, sub-score systolic blood pressure (**C**–**D**), COMPASS 31 questionnaire with LF-sBP (**E**); orthostatic intolerance sub-sore and LF-sBP (**F**), COMPASS 31 score with LFnu-sBP (**G**); orthostatic intolerance sub-sore with LFnu-sBP (H)

For MG group, median COMPASS-31 total score was 31.8 [18.8–49.4]. Patients with moderate MG showed higher overall COMPASS-31 score (*p*=0.031) and OI sub-score (*p*=0.019) compared with mild MG patients (Table 1). OI symptoms (OI sub-score>0) were reported by the 72% of the MG patients (88.9% moderate, 62.5% mild). In MG patients, the total COMPASS-31 score showed inverse correlation with sBP, mBP (*R*=−0.28, *p*=0.048) at rest, and sBP, dBP (*R*=−0.32, *p*=0.022), mBP (*R*=−0.34, *p*=0.014), LFnu-sBP, LF-sBP during HUTT. Similarly, the OI score was inversely correlated with values of sBP at rest and sBP, LFnu-sBP, LF-dBP (*R*=−0.29, *p*=0.044) during HUTT and positively correlated with MGFA (*R*=0.30, *p*=0038) (Fig. 2).

## Discussion

To the best of our knowledge, this is the first study that investigates very short-term BPV in a relatively large group of clinically stable MG patients. The main finding of this study is that MG patients present significant alterations in BPV, both at rest and in response to orthostatic stress, which are related to autonomic symptoms and disease severity. Furthermore, a higher overall COMPASS-score and OI sub-score were related to lower values of blood pressure at rest and during HUTT, as well as lower low frequency of BPV parameters during HUTT.

Our results confirm and extend prior studies that non-invasive tool like BPV is promising source of additional information about cardiovascular autonomic function, particularly the sympathetic activity and status of the sympathovagal balance, in the course of MG disease [26]. We demonstrate that moderate MG patients were characterized by an overall shift of sympathovagal balance toward sympathetic predominance as shown by increase LF/HF-dBP, either at rest and during HUTT, compared to HCs and mild MG patients as well as higher values of resting LFnu-DBPV and LF/HF, compared to mild MG. Given that the LF component of DBPV is considered index of efferent sympathetic vascular modulation, it is reasonable to assume that the increase in LF power may suggest enhanced regulatory function of resting sympathetic vasomotor activity in the moderate MG group [16].

Another interesting finding in our study is that, compared to HCs and mild MG, moderate MG patients were characterized by lower values of HFnu, of DBPV during HUTT, as well as lower post tilt changes in ΔHFnu of SBPV than HCs, suggesting perhaps less parasympathetic withdrawal in response to HUTT and/or respiratory influences. Previous studies have shown that HF component of BPV is strongly influenced by intrathoracic pressure and mechanical effects of breathing on blood pressure changes [16]. Along these lines, moderate MG patients had higher respiratory rate compared to HCs and mild MG patients. Considering that MG is characterized by altered ventilatory pattern and respiratory muscle weakness, this data suggests a possible effect of respiration on HF-BPV.

Consistent with previous studies, both MG subgroups have relatively preserved sympathetic functioning in response to orthostatic challenge [7, 9, 10], with however lower ΔdBP, ΔmBP in mild MG patients, as compared to HCs. Lower blood pressure response during HUTT in our mild MG patients most likely resulted from lower values of LFnu-dBP, and/or lower sympathovagal balance at rest position. Interestingly, orthostatic intolerance symptoms, including POTS in two patients with mild and OH in one patient with moderate MG, could be attributed to mild dysautonomia. These observations are in line with previous studies, suggesting presence of early manifestations of cardiovascular autonomic dysregulations, even in mild stage of the MG disease [11].

Concerning the frequency of overall AD in MG subgroups, moderate MG patients had higher overall COMPASS-31 score and OI sub-score suggesting greater autonomic symptom burden [17, 18]. About one-third (72%) of MG patients have demonstrated OI symptoms, which is in line with the high prevalence (80%) reported by Benjamin et al. among 17 MG patients with myasthenic crisis. They also found that one patient had features of POTS during tilt [27]. Limited evidence suggests the possibility of sympathetic deficiency of myasthenic patients [11, 13]. In line with this, in our study higher overall COMPASS-score and OI sub-score were related to a higher disease severity, lower values of blood pressure (sBP, dBP) at rest and during HUTT, as well as and lower values of low frequency of BPV parameters during HUTT. The strongest, but moderate association was observed between OI sub-score and sBP during orthostatic challenge. Together with BPV results, these observations highlight possible development of ANS dysregulation, including greater OI symptoms with disease progression.

Importantly, the association between MG and cardiac autonomic dysfunction was previously documented and thought to underlie the increased risk of incidence of cardiovascular events including atrial fibrillation, arrythmia, syncope [6]. In this context, inability to raise the diastolic blood pressure during orthostasis may increase risk for episodes of myocardium hypoperfusion, particularly in the presence of coronary artery disease [28]. Sympathetic hyperactivity is considered a significant emerging risk factor for increase in end-organ damage as well as cardiovascular mortality [24]. A recent case-control study with 1660 MG patients demonstrated that more than 78.7% of all study participants reported at least one comorbid disease with cardiovascular diseases being the most common one (37%) followed by other autoimmune diseases (23.7%) [29].

Moreover, our BPV results are in some extent consistent with our previous observations derived from cardiovascular reflex tests, HRV, and BRS method, suggesting sympathovagal imbalance in favor of sympathetic tone, lower cardiovagal tone, and lower left ventricular myocardial function in patients with MG compared to HCs [9].

We cannot rule out that these BPV differences are to some extent due to administration of medications, as described in previous studies [7, 10]. In fact, all of our patients had been on acetylcholinesterase inhibitors, and 43% of them used immunosuppressive agents. Previous studies have demonstrated that chronic cholinergic stimulation with pyridostigmine bromide promotes benefits, enhances vagal tonus, reduces orthostatic symptoms, increased HRV oscillations variability (HRV) and baroreflex sensitivity (BRS), indicating an improvement in cardiac autonomic control [30]. In another study performed using hypertensive rats, cholinergic stimulation significantly reduced SBPV variability and increased vagal influence on cardiac autonomic balance. [30]. These reports confirm our findings, especially in relation to the mild MG group. Moreover, we observed that higher proportion of the moderate MG patients was administered steroid and immunosuppression therapy often administered in more advanced stages of disease. Four of our mild patients and 10 of moderate MG patients were taking combined immunosuppression therapy; thus, it was not possible to examine the effects of free immunosuppression in our cohort.

There is still debate on whether changes in ANS in MG could be a result of disease itself or whether chronic inflammation process influences ANS imbalance. There are several factors that can affect ANS imbalance in MG One of these factors is disrupted cholinergic transmission in autonomic ganglia [31. The ganglionic (α3-type) neuronal AChR mediates fast synaptic transmission in sympathetic, parasympathetic, and enteric autonomic ganglia. Experimental evidence indicates that the ganglionic AChR is structurally similar to the muscle AChR at the neuromuscular junction and contains the neuronal α3-AChR subunit most commonly associated with the β4 subunit. Vernino et al. described seven patients with MG and dysautonomia and found that autonomic dysregulation might be a result of humoral autoimmunity against ganglionic AChRs [31]. Watari et al. found that anti*-*ganglionic nicotinic acetylcholine receptor (gAChR) antibodies occurred more frequently in patients with POTS compared to HCs, suggesting that anti-gAChR antibodies may be associated with POTS and its underlying autonomic [32].

Systemic inflammation is also a plausible factor that leads to ANS imbalance and BPV alterations. Although MG mainly affects the neuromuscular junction in the periphery outside of central nervous system, a study of Huang et al. showed the systemic inflammation markers expressed abnormally in MG patients [33]. Sympathetic nervous system (SNS) exhibits a complex bidirectional influence on inflammatory state, suggesting that on the systemic level the activation of the SNS can trigger or suppress the activity of immune cells [34]. In this regard, Uzawa and colleagues found that both anti-inflammatory and inflammatory cytokines are upregulated in MG, reflecting the importance of cytokine-mediated inflammation and its regulation in MG pathophysiology [35].

SNS regulates vasomotor tone by norepinephrine binding α1-adrenergic receptors (α1-ARs) on smooth muscle, resulting in contraction, increased vascular resistance, and elevated blood pressure [36]. Indeed, we recently reported that MG patients exhibit increased vasomotor tone, expressed as a higher resting total peripheral resistance index when compared to healthy controls [9]. Although α1-ARs are less abundant in immune cells, there is evidence indicating that α1-adrenergic receptors might contribute to the amplification of cytokine secretion in innate cells T [36]. Moreover, activation of α-ARs is generally associated with pro-inflammatory functions, while β adrenergic receptors, especially β2 receptors, are related to the resolution of inflammation and tissue remodeling [34]. Circulating inflammatory cytokines can also induce vasoconstriction and impair endothelium-dependent vasodilation. These effects may contribute to vessel spasm and endothelial dysfunction, further strengthening the connection between inflammation and vascular disease [37]. Taken together, this viewpoint may possibly account for enhanced resting sympathetic vasomotor activity in moderate MG patients, as shown by LFnu-dBP. There are only few studies investigating BP response in patients with MG. Nikolic et al. compared 27 AChR-positive patients (MGFA I-IIIb) with and 25 AChR-positive without thymoma (MGFA I-IIIb), and 23 MuSK patient (MGFA I-IIIb) to HCs, and confirm autonomic impairment in different forms of MG. They found that patients with thymoma-associated MG presented moderate failure in both sympathetic and parasympathetic branch of the ANS, using Ewing battery of tests (handgrip test, orthostatic challenge). However, when they analyzed ANS dysfunction in the patients with AChR-positive MG without thymoma they found mild ANS dysfunction, with no signs of sympathetic dysfunction in comparison to HCs, which is in line with our results presented in both MG subgroups. In contrast, Puneeth et al. compared 30 mild MG patients (4 patients in remission, 25 patients with Osserman grade I–IIa) with 30 controls and reported higher resting LF/HF ratio and significant decrease in values of blood pressure using isometric handgrip test in patients group, suggesting significant parasympathetic deficiency and minimal sympathetic deficiency. Another study showed that MG patients (12 patients in remission, 15 with mild MGFA, 3 moderate to severe MGFA) compared to controls have significantly more pronounced sympathetic activity at rest and during tilt expressed as higher LF/HF ratio as well as both systolic and diastolic blood pressure [10]. Similarly, Shukla et al. in study of 64 MG patients with thymoma showed significantly higher rise in HR and BP using Ewing battery tests (orthostatic tests, isometric hand grip test) compared to HCs. The discrepancy between our results is most likely related to differences in sample sizes, ANS evaluation, and stratification of MG disease; thus, these findings require further study.

The relationship between BPV and disease phenotype has also been investigated in patients with multiple sclerosis (MS) [38-40]. For example, Crnošija et al. [38] found significant differences in SBPV parameters between the patients with secondary progressive MS (SPMS), clinically isolated syndrome (CIS), and healthy controls. They observed that individuals with SPMS had diminished sympathetic vasomotor activity compared to the CIS and healthy control groups, both at rest and in response to tilt. However, CIS patients did not show significant differences compared to healthy controls. This suggests that lower LF SBPV in SPMS patients may be due to desensitization of peripheral adrenergic receptors resulting from a chronically overactive SNS. In another study involving progressive MS patients (including primary progressive and secondary progressive variants), higher values of low-frequency normalized units of systolic blood pressure (LFnu-sBP) at rest were observed compared to relapsing-remitting MS and healthy control groups. Additionally, higher disease severity, as indicated by an Expanded Disability Status Scale (EDSS) score, was associated with decreased post-tilt changes in LF/HF-sBP, indicating progressive deterioration of sympathetic modulation [39]. Furthermore, a higher EDSS score and male sex may also be considered significant predictors of dBP increase for MS patients [40]. Further research is necessary to establish a clearer understanding of the relationship between BPV and autoimmune diseases.

Nonetheless, there are several limitations. First, selection biases limit the representativeness of our sample. Because our analyses included patients who were in I-III MGFA and none of them was classified as subclass B, our findings are not generalizable to other populations, including more severe individuals as well as those with predominant bulbar-oropharyngeal muscles involvement. Second, our study may not have investigated all of the autonomic function, such as BRS, HRV, cardiovascular reflex tests; however, these alterations we explored in previous studies [9, 22]. Third, age, medication taking, low physical activity, hydration status can be said to act as confounding variables and as effect modifiers.

## Conclusions

MG patients present significant alterations in BPV, both at rest and in response to orthostatic stress, which are related to autonomic symptoms and disease severity. Our observations highlight possible development of ANS dysregulation, including greater OI symptoms with disease progression. Moreover, these findings emphasize the importance of monitoring BPV when evaluating cardiovascular autonomic function and its evolution over the course of MG disease.

## References

[1] Gilhus NE, Tzartos S, Evoli A (2019). Myasthenia gravis. Nat Rev Dis Primers.

[2] Melzer N, Ruck T, Fuhr P (2016). Clinical features, pathogenesis, and treatment of myasthenia gravis: a supplement to the Guidelines of the German Neurological Society. J Neurol.

[3] Howard JF (2018). Myasthenia gravis: the role of complement at the neuromuscular junction. Ann N YAcad Sci.

[4] Suzuki S, Utsugisawa K, Suzuki N (2013). Overlooked non-motor symptoms in myasthenia gravis. J Neurol Neurosurg Psychiatry.

[5] Tajima Y, Yaguchi H, Mito Y (2019). Non-motor comorbidity of myasthenia gravis: myasthenia gravis as a systemic immunological disorder involving non-motor systems. Intern Med.

[6] Shivamurthya P, Parkerb MW (2014). Cardiac manifestations of myasthenia gravis: a systematic review. IJC Metab Endocr.

[7] Peric S, Rakocevic-Stojanovic V, Nisic T (2011). Cardiac autonomic control in patients with myasthenia gravis and thymoma. J Neurol Sci.

[8] Nikolić A, Perić S, Nišić T (2014). The presence of dysautonomia in different subgroups of myasthenia gravis patients. J Neurol.

[9] Zawadka-Kunikowska M, Rzepiński Ł, Tafil-Klawe M (2022). Association of cardiac autonomic responses with clinical outcomes of myasthenia gravis: short-term analysis of the heart-rate and blood pressure variability. J Clin Med.

[10] Kocabas ZU, Kizilay F, Basarici I (2018). Evaluation of cardiac autonomic functions in myasthenia gravis. Neurol Res.

[11] Puneeth CS, Chandra SR, Yadav R (2013). Heart rate and blood pressure variability in patients with myasthenia gravis. Ann Indian Acad Neurol.

[12] Shukla G, Gupta S, Goyal V, Singh S, Srivastava A, Behari M (2013). Abnormal sympathetic hyper-reactivity in patients with myasthenia gravis: a prospective study. Clin Neurol Neurosurg.

[13] Stoica E, Enulescu O (1992). Deficiency of sympathetic nervous system function in myasthenia. J Auton Nerv Syst.

[14] Low PA (1993). Composite autonomic scoring scale for laboratory quantification of generalized autonomic failure. Mayo Clin Proc.

[15] Cheshire WP, Freeman R, Gibbons CH (2021). Electrodiagnostic assessment of the autonomic nervous system: a consensus statement endorsed by the American Autonomic Society, American Academy of Neurology, and the International Federation of Clinical Neurophysiology. Clin Neurophysiol.

[16] Pagani M, Lucini D, Rimoldi O, Furlan R (1996). Low and high frequency components of blood pressure variability. Ann N Y Acad Sci.

[17] Foschi M, Giannini G, Merli E, Mancinelli L, Zenesini C, Viti B, Guaraldi P, Cortelli P, Lugaresi A (2021). Frequency and characteristics of dysautonomic symptoms in multiple sclerosis: a cross-sectional double-center study with the validated Italian version of the Composite Autonomic Symptom Score-31. Neurol Sci.

[18] Hilz MJ, Wang R, Singer W (2022). Validation of the Composite Autonomic Symptom Score 31 in the German language. Neurol Sci.

[19] Barnett C, Herbelin L, Dimachkie MM (2018). Measuring clinical treatment response in myasthenia gravis. Neurol Clin.

[20] Jaretzki A, Barohn RJ, Ernstoff R (2000). Myasthenia gravis: recommendations for clinical research standards. Task force of the medical scientific advisory board of the myasthenia gravis foundation of America. Neurology.

[21] Rzepiński Ł, Zawadka-Kunikowska M, Newton JL (2021). Cardiac autonomic dysfunction in myasthenia gravis and relapsing-remitting multiple sclerosis—a pilot study. J Clin Med.

[22] Therapeutics and Technology Assessment Subcommittee of the American Academy of Neurology (1996). Assessment clinical autonomic testing report of the therapeutics and technology subcommittee of the American Academy of Neurology. Neurology.

[23] Parati G, Ochoa JE, Bilo G (2012). Blood pressure variability, cardiovascular risk, and risk for renal disease progression. Curr Hypertens Rep.

[24] Bianchi AM, Mainardi L, Meloni C (1997). Continuous monitoring of the sympatho-vagal balance through spectral analysis. IEEE Eng Med Biol Mag.

[25] NIST/SEMATECH e-Handbook of statistical methods. Available online: http://www.itl.nist.gov/div898/handbook/.2012/01/20 (accessed on 21 August 2020)

[26] Laitinen T, Hartikainen J, Niskanen L (1999). Sympathovagal balance is major determinant of short-term blood pressure variability in healthy subjects. Am J Physiol.

[27] Benjamin RN, Aaron S, Sivadasan A, Devasahayam S, Sebastin A, Alexander M (2018). The spectrum of autonomic dysfunction in myasthenic crisis. Ann Indian Acad Neurol.

[28] Wijkman M, Länne T, Östgren CJ (2016). Diastolic orthostatic hypertension and cardiovascular prognosis in type 2 diabetes: a prospective cohort study. Cardiovasc Diabetol.

[29] Lehnerer S, Jacobi J, Schilling R (2022). Burden of disease in myasthenia gravis: taking the patient’s perspective. J Neurol.

[30] Soares PP, da Nóbrega AC, Ushizima MR (2004). Cholinergic stimulation with pyridostigmine increases heart rate variability and baroreflex sensitivity in rats. Auton Neurosci.

[31] Vernino S, Cheshire WP, Lennon VA (2001). Myasthenia gravis with autoimmune autonomic neuropathy. Auton Neurosci.

[32] Watari M, Nakane S, Mukaino A (2018). Autoimmune postural orthostatic tachycardia syndrome. Ann Clin Transl Neurol.

[33] Huang X, Xu M, Wang Y (2023). The systemic inflammation markers as possible indices for predicting respiratory failure and outcome in patients with myasthenia gravis. Ann Clin Transl Neurol.

[34] Pongratz G, Straub RH (2014). The sympathetic nervous response in inflammation. Arthritis Res Ther.

[35] Uzawa A, Kanai T, Kawaguchi N (2016). Changes in inflammatory cytokine networks in myasthenia gravis. Sci Rep.

[36] Sharma D, Farrar JD (2020). Adrenergic regulation of immune cell function and inflammation. Semin Immunopathol.

[37] Vila E, Salaices M (2005). Cytokines and vascular reactivity in resistance arteries. Am J Physiol Heart Circ Physiol.

[38] Crnošija L, Moštak I, Višnjić N, Junaković A, Karić A, Adamec I, Krbot Skorić M, Habek M (2022). Blood pressure variability is altered in secondary progressive multiple sclerosis but not in patients with a clinically isolated syndrome. Neurophysiol Clin.

[39] Zawadka-Kunikowska M, Rzepiński NJL (2020). Cardiac autonomic modulation is different in terms of clinical variant of multiple sclerosis. J Clin Med.

[40] Rzepiński Ł, Zawadka-Kunikowska M, Newton JL (2022). Cardiovascular autonomic dysfunction in multiple sclerosis—findings and relationships with clinical outcomes and fatigue severity. Neurol Sci.

